# Relationship of prenatal maternal obesity and diabetes to offspring neurodevelopmental and psychiatric disorders: a narrative review

**DOI:** 10.1038/s41366-020-0609-4

**Published:** 2020-06-03

**Authors:** Linghua Kong, Xinxia Chen, Mika Gissler, Catharina Lavebratt

**Affiliations:** 1 Department of Molecular Medicine and Surgery, Karolinska Institutet, Stockholm, Sweden; 2 Center for Molecular Medicine, Karolinska University Hospital, Stockholm, Sweden; 3 School of Nursing, Shandong University, Shandong, China; 4 National Institute for Health and Welfare, Helsinki and Oulu, Oulu, Finland; 5 Division of Family Medicine, Department of Neurobiology, Care Sciences and Society, Karolinska Institutet, Stockholm, Sweden; 6 University of Turku, Research Centre for Child Psychiatry, Turku, Finland

**Keywords:** Obesity, Pre-diabetes, Obesity, Pre-diabetes

## Abstract

Obesity and diabetes is a worldwide public health problem among women of reproductive age. This narrative review highlights recent epidemiological studies regarding associations of maternal obesity and diabetes with neurodevelopmental and psychiatric disorders in offspring, and provides an overview of plausible underlying mechanisms and challenges for future human studies. A comprehensive search strategy selected terms that corresponded to the domains of interest (maternal obesity, different types of diabetes, offspring cognitive functions and neuropsychiatric disorders). The databases searched for articles published between January 2010 and April 2019 were PubMed, Web of Science and CINAHL. Evidence from epidemiological studies strongly suggests that maternal pre-pregnancy obesity is associated with increased risks for autism spectrum disorder, attention-deficit hyperactivity disorder and cognitive dysfunction with modest effect sizes, and that maternal diabetes is associated with the risk of the former two disorders. The influence of maternal obesity on other psychiatric disorders is less well studied, but there are reports of associations with increased risks for offspring depression, anxiety, schizophrenia and eating disorders, at modest effect sizes. It remains unclear whether these associations are due to intrauterine mechanisms or explained by confounding family-based sociodemographic, lifestyle and genetic factors. The plausible underlying mechanisms have been explored primarily in animal models, and are yet to be further investigated in human studies.

## Introduction

Obesity among women of reproductive age increased globally from 2005 to 2014, although the rates varied widely between countries [1]. More than 30% of U.S. women aged 20-39 years were defined as obese (BMI≥30 kg/m^2^) in 2011-2014 [2]. The rise in the prevalence of obesity in this group is deemed as a major determinant of an increased risk of type 2 diabetes mellitus (T2DM) and gestational diabetes mellitus (GDM). Diabetes is characterized by elevated blood glucose levels, impaired insulin secretion, and/or peripheral resistance to insulin action. A systematic literature review reported in 2013 that worldwide up to 16.0% of women in reproductive age (20–49 years) were affected in pregnancy by diabetes, and the highest prevalence was found in the South-East Asia Region at 25.0% while in the North America and Caribbean Region the prevalence was 10.4% [3]. Around 87.5% of maternal diabetes cases are GDM, 7.5% are pre-pregnancy type 1 diabetes mellitus (T1DM), and 5% are T2DM [4]. Moreover, the prevalence of maternal diabetes is steadily increasing [5].

Maternal obesity has adverse effects on fetal outcomes, such as prematurity and congenital anomalies, as well as increases the risk for offspring adiposity, and disorders of offspring cardio-metabolism, respiration and cognition [6–9]. Besides, maternal obesity are reported to increase risks for offspring neuropsychiatric disorders, such as autism spectrum disorder (ASD), attention deficit hyperactivity disorder (ADHD), anxiety and depression, schizophrenia and other neuropsychiatric disorders [10] (Table 1). While the association between maternal obesity and offspring neuropsychiatric disorder has been extensively studied, especially for ASD, the influence of maternal diabetes on offspring neuropsychiatric disorders, other than ASD and ADHD [11–16], is less well explored. Likewise, little is known regarding the size of the joint effects of maternal pre-pregnancy obesity and different types of maternal diabetes on neuropsychiatric outcomes in offspring [12]. Maternal obesity is highly comorbid with T2DM and GDM [17], and proposed mechanisms of their associations with offspring neuropsychiatric disorders overlap between obesity and diabetes [18].

**Table 1:** Human epidemiological studies examining effects of maternal obesity and diabetes on offspring neurodevelopmental and psychiatric disorders.

Offspring morbidity	Specific outcomes	Maternal exposures	Adjusted factors	Sample	Study design	Finding	Reference
Neuropsy-chiatric disorders	The diagnosis groups indicated by ICD-10 codes: F80 to F83, F84, F90 to F91, F92 to F95, F98, F20- F45, F50, and F51.	Pre-pregnancy BMI and PGDM	I:1,2,10,11 II:1,2,3,4, 7,9,11,13	All live births in Finland between 2004 and 2014 (*n* = 649,043)	Cohort (Finland)	Maternal PGDM combined with severe maternal obesity markedly increased the risk of several children’s psychiatric and mild neurodevelopmental disorders.	Kong et al. [12]
	The ADHD-DSM-IV form was completed by teachers. Cognitive and motor development tests were performed at the primary health center.	Pre-pregnancy BMI	I:1,2,5,7, 12 II:1,5,6,9,11 III:1,6	1827 Spanish children	Cohort (Spain)	Maternal obesity was associated with a reduction in offspring verbal scores at pre-school age.	Casas et al. [30]
	Children cognitive and psychomotor development were assessed at around age 14 months (range 11–22 months).	Pre-pregnancy BMI	I:2,4,5,12 II:1,3,4,5, 6,9 III:1,3,5,6	2644 mothers were recruited and 2226 children from four Spanish regions between 2004 and 2008	Cohort (Spain and Greece)	Maternal obesity was associated with reduced child cognitive development at early ages.	Casas et al. [44]
	Children’s mental and motor development were assessed by the Bayley Scales of Infant Development-II (BSID-II) and the Bayley Short Form-Research Edition (BSF-R) at age 2 years.	Pre-pregnancy BMI	I: 2,3,5 II:1,2,3,4, 5,6,9,10	6850 US children born in 2001	Cohort (USA)	An association was found between maternal BMI status and children’s mental development but no association with children’s motor development.	Hinkle et al. [45]
ASD	ASD and other DDs were based on physician diagnoses as documented in electronic medical records.	Pre-pregnancy obesity, GDM	I:1,2,9 II:1,4,9	2734 children using a subset of the Boston Birth Cohort between 1998 and 2014	Cohort (USA)	Maternal obesity and maternal diabetes in combination were associated with increased risk for ASD and ID. ASD with ID may be etiologically distinct from ASD without ID.	Li et al. [15]
	ASD based on pediatric developmental specialist evaluations.	Pre-pregnancy BMI	I:1,2,3 II:1,3,4,5, 6,9,10,12, 13,14	322,323 singleton children born in 1995-2009 at Kaiser Permanente Southern California (KPSC) hospitals	Cohort (USA)	Exposure to maternal GDM diagnosed by 26 weeks’ gestation was associated with risk of ASD in offspring.	Xiang et al. [14]
	ASD diagnosis using ICD-9, ICD-10, and DSM-IV codes.	Pre-pregnancy BMI, GWG	I:1,2,11 II:1,3,6,11 III:3,5,7	333,057 individuals born 1984–2007, of whom 6,420 with ASD	Cohort (Sweden)	Maternal BMI was associated with ASD. Sibling analyses and paternal BMI analyses indicated that maternal BMI may also be a proxy marker for other familial risk factors.	Gardner et al. [28]
	ASD diagnostic from test and interview results and from information collected from parents and teachers.	Pre-pregnancy BMI	I:1 II:5,9 III:1	92,909 children born from 1999 to 2009	Cohort (Norway)	Both maternal and paternal obesity were associated with an increased risk of ASD in offspring.	Surén et al. [29]
	ASD diagnosis identified by trained clinicians.	Maternal diabetes, hypertension, and obesity during pregnancy	I:1,2,4 II:1,3,5,	Children aged 2 to 5 years (517 ASD, 172 DD, and 315 controls)	Case-control study (USA)	Diabetes, hypertension, and obesity were more common among mothers of children with ASD compared with controls. Diabetes, in particular, was associated with statistically significantly greater deficits in expressive language among children with ASD.	Krakowiak et al. [27]
	ASD identified by questionnaire and parental report.	GDM	I:8 II:1,2,3,6, 8,9,13 III:1,5	116,608 female nurses aged 25–42 years when recruited in 1989	Cohort (USA)	GDM was associated with an increased risk of ASD in offspring.	Lyall et al. [32]
	ASD diagnosis identified using ICD-9 code 299 or ICD-10 code F84.	Pre-pregnancy weight and GWG	I:1,2,5,13 II:7,10,11	129,733 children born between 1990 and 2002	Cohort (Canada)	Maternal weight of 90 kg or more and weight gain of 18 kg or more were both independent risk factors for ASD.	Dodds et al. [33]
ADHD	ADHD cases were identified based on ICD-9 codes 314 or refills of ADHD-specific medications during the follow-up window from at least two separate visits.	T1DM, T2DM, GDM	I:1,2,11 II:1,3,5,6, 9,11,13	333,182 singletons born in 1995–2012	Cohort (USA)	Compared with children unexposed to diabetes, the adjusted HRs for ADHD in children were 1.57 (95% CI 1.09–2.25) for exposure to T1DM, 1.43 (1.29–1.60) for T2DM, 1.26 (1.14–1.41) for GDM requiring antidiabetic medications, and 0.93 (0.86– 1.01) for GDM not requiring medications.	Xiang et al. [13]
	Individuals with ADHD were identified according to ICD-9 code 314 and ICD-10 code F90.	T1DM	I:1,2,6,10 II:1,4,5,6, 11,13 III:1,3,5,7	15,615 individuals were born after parental diagnosis of T1DM, and 1,380,829 children with parents without a diagnosis of T1DM were matched control subjects	Cohort (Sweden)	In this retrospective cohort study, we found that a parental history of T1D was associated with a 29% increased risk of being diagnosed with ADHD.	Ji et al. [40]
	ADHD diagnosis (ICD-10 codes F90.0, F90.1, or F98.8) and ASD diagnosis (ICD-10 code F84.0, F84.1, F84.5, F84.8, or F84.9).	Pre-pregnancy BMI	I:3 II:1,4,6,11,14 III:5	81,892 children of whom 2417 (3.0%) had an ADHD diagnosis, and 1118 (1.4%) had an ASD diagnosis while 606 children (0.7%) had both disorders	Cohort (Denmark)	An association was found between maternal overweight and increased risk of ADHD, With regard to ASD, there was an elevated risk of ASD in children with underweight or obese mothers.	Andersen et al. [170]
	ADHD diagnosis (ICD-10 codes F90 + F98.8).	Auto-immune diseases including T1DM	I:1,2 II:11 III:7	All children born in Denmark from 1990 to 2007 (*N* = 983,680).	Cohort (Denmark)	A personal history and a maternal history of autoimmune disease were associated with an increased risk of ADHD. The previously reported association between	Nielsen et al. [39]
						T1DM and ADHD was confirmed.	
	ADHD diagnosis (ICD-9 codes 314.00 or 314.01) at age 5–12 years.	Pre-pregnancy BMI	I:1,2,11 II:1,10,11,14	4682 (*N* = 187 with ADHD) children born to 3645 different mothers.	Sibling-compari-son design (USA)	The association between maternal pre-pregnancy BMI and offspring ADHD may be better accounted for by familial or maternal confounds rather than by a direct effect of maternal BMI.	Musser et al. [37]
	The dispensed and reimbursed ADHD medications methylphenidate (ATC code N06BA04), atomoxetine (ATC code N06BA09), and racemic amphetamine (ATC code N06BA01).	Inflamma-tory and immune system diseases (TIDM, T2DM, hyperten-sion)	I:1,3 II:1,2,5,9, 12,14 III: 8	All individuals (*N* = 2,322,657) born during the period 1967–2008	Register-based case-control study (Denmark)	TIDM was associated with ADHD in offspring. In contrast, chronic hypertension and T2DM were not associated with ADHD.	Instanes et al. [38]
	Hyperkinetic disorder diagnoses (ICD-9: 314; ICD-10: F90); or ADHD (DSM-IV-TR: 314; ICD-10: F90); or treated with ADHD medication at age 3 years.	Pre-pregnancy BMI	I:1,2,11 II:1,3,4,5, 15	673,632 individuals born in Sweden between 1992 and 2000	Cohort (Sweden)	At the population level, maternal overweight and obesity were associated with increased risk of offspring ADHD. In full sibling comparisons, however, previously observed associations no longer remained.	Chen et al. [36]
	ADHD diagnosis rated using DSM-IV.	GDM	I:1,2,3,4 II:1,4,11 III:7	*N* = 212; 3 and 4-year-old children in preschools	Cohort (USA)	GDM and low socioeconomic status, especially in combination, heighten the risk for childhood ADHD.	Nomura et al. [11]
Cognitive Function/ Intellectual Disability	IQ was assessed using Stanford Binet Intelligence Scale–4th edition. Executive function was assessed by the number of perseverative errors on the Wisconsin Card Sorting Test and time to complete Part B on the Trail Making Test.	Pre-pregnancy BMI and GWG	I:2, II:3,6,9,11,12,	Mother–infant dyads (*n* = 763) enrolled in a birth cohort study were followed from early pregnancy to 10 years postpartum	Cohort (USA)	Although GWG may be important for executive function, maternal BMI has a stronger relation than GWG to both offspring intelligence and executive function.	Pugh et al. [46]
	Educational achievement at age 16 years and IQ at the conscription examination at 18 years of age.	Diabetes in pregnancy	I:1,3 II:1,5,9,10,14	Sibling study with 723,775 men from 579,857 families in Sweden	Cohort (Sweden)	Diabetes associated with lower educational achievement and lower IQ.	Fraser et al. [53]
	IQ was measured by Wechsler Intelligence Scales at 7 years of age.	Pre-pregnancy BMI	II:1,2,3,4, 5,6,9	30,212 children born to US mothers between 1959 and 1965	Cohort (USA)	Maternal obesity was associated with lower child IQ (2–2.5 points lower), and excessive weight gain accelerated the association (6.5 points lower).	Huang et al. [50]
	IQ was assessed with the Wechsler Primary and Preschool Scales of Intelligence – Revised (WPPSI-R).	Pre-pregnancy BMI	I:1,2,3,5 II:1,2,4,5, 9,10,14 III:1,6	1,783 mothers and their 5-year-old children sampled from the Danish National Birth Cohort	Cohort (Denmark)	Both maternal and paternal BMI were associated with lower IQ.	Bliddal et al. [43]
	Cognitive skills were assessed using the PIAT mathematics and reading recognition scores.	Pre-pregnancy BMI	I:2,3,4,11 II:1,5,6,10,11	3,412 US children aged 60 to 83 months between 1986 and 2008.	Observa-tional design (USA)	Maternal obesity was associated with reductions in child cognitive test scores (reading recognition and mathematics).	Tanda et al. [47]
	Cognitive performance was assessed using British Ability Scales (BAS-II) at ages 5 or 7 years.	Pre-pregnancy BMI	I:2,3,4 II:1,4,5,6, 13 III:1,3	11,025 children at 5 years, and 9,882 children at 7 years in the United Kingdom	Cohort (UK)	Maternal BMI was associated with lower cognitive performance. The relationship appears to become stronger as children get older.	Basatemur et al. [42]
	Dataset A: assessed intelligence using The Bayley Scales of Infant Development (BSID-III). Dataset B: assessed intelligence with the Wechsler Intelligence Scale for Children (WISC III).	BMI during pregnancy	I:2,11 II:1,4	Dataset A: 5,734 women between 2004 and 2006; Dataset B: 25,216 women between 1987 and 1990.	Cohort (USA)	Second trimester maternal obesity may be an independent risk factor for some aspects of children’s neurocognitive development.	Craig et al. [49]
	School Entry Assessment (SEA) results (age 4 years), IQ (age 8 years), and General Certificate of Secondary Education (GCSE) results (age 16 years).	GDM, preexist-ing diabetes	I:2,5 II:1,4,5,6, 7,9,10,14	8515 women from the Avon Longitudinal Study of Parents and Children (ALSPAC) delivered between 1991 and 1992	Cohort (UK)	Maternal diabetes in pregnancy was associated with lower offspring cognition and educational attainment.	Fraser et al. [51]
	Cognitive function assessed by Raven’s Standard Progressive Matrices and three verbal subtests (information, similarities and vocabulary) from the Weschler Adult Intelligence Scale (WAIS)	T1DM	I:1,2,3 II:1,4,5,6, 9,13,14 III:1,3	Adult offspring of women with Type 1 diabetes (*n* = 158) and a reference group from the background population (*n* = 118)	Cohort (Denmark)	Impaired cognitive function in adult offspring of women with T1DM. Harmful effects of maternal hyperglycemia may be mediated through delivery at <34 weeks.	Clausen et al. [54]
	Children’s cognition and behavior was assessed by parents.	Pre-pregnancy BMI	II:4,5,6 III:1,3	British ALSPAC (*N* = 5000) and Dutch Generation R (*N* = 2500) cohorts	Cohort (UK and Netherlands)	Little consistent evidence of intrauterine effects of maternal overweight on child cognition and behavior.	Brion et al. [48]
	Cognitive function assessment using tests from the Kaufman Assessment Battery for children-second edition and additional tests measuring learning, long-term storage/retrieval, short-term memory, reasoning, attention and concentration, visio-spatial and verbal abilities.	GDM	I:1,2,3 II:1,3,5,6, 9,10 III:1,3	515 healthy children (32-offspring of mothers with GDM; 483 offspring of non-GDM mothers (controls) from the Mysore Parthenon birth cohort	Cohort (India)	There was no evidence of lower offspring cognitive ability for mothers with GDM.	Veena et al. [52]
Affective disorders	Affective problems were assessed using the CBCL/4–18 according to DSM-IV.	Pre-pregnancy BMI	I:3,5 II:1,4,5,6, 13,14,15 III:3,6	2868 live-born children in the 17-year follow-up since 1989	Cohort (Australia)	Overweight or obesity were associated with increased risks for affective problems during childhood and adolescence.	Robinson et al. [56]
Psychosis and schizophrenia	Non-affective psychoses was assessed by ICD-10: F20-F29; ICD-9 codes 295, 297 and 298, except 298A and 298B; and narrowly defined schizophrenia (ICD-9 code 295 and ICD-10 code F20).	Pre-pregnancy BMI, GWG	I:1,2,11 II:1,3,6,11 III:3,4,5,7	Follow-up 526,042 children born between 1982 and 1989	Cohort (Sweden)	A weak U-shaped association between maternal BMI at the beginning of pregnancy and the increased risk for non-affective psychosis in offspring. No association was apparent between elevated maternal BMI and schizophrenia risk.	Mackay et al. [58]
Eating Disorders	Diagnosis of eating disorder (anorexia, bulimia, and binge-eating disorder) by DSM-IV or DSM-V criteria.	Pre-pregnancy BMI	No adjusted factors	*N* = 1383; population-based sample of male and female adolescents followed from ages 14–20 years	Cohort (Australia)	Each one-unit increase in maternal early pregnancy BMI increased odds of eating disorders in offspring by 10%.	Allen et al. [59]

I. Child factors: 1 = birth year, 2 = birth sex, 3 = birth weight (BMI or child’s weight and head circumference at birth), 4 = race/ethnicity, 5 = breastfeeding duration, 6 = low Apgar score, 7 = child physical activity, 8 = twin births, 9 = preterm birth, 10 = small for gestational age, 11 = birth order, 12 = nursery attendance and/or main childminder, 13 = sibling with neuropsychiatric disorders.

II. Maternal factors: 1 = age at delivery, 2 = marital status, 3 = race/ethnicity, 4 = smoking and/or alcohol use, 5 = education, 6 = socioeconomic status, 7 = mode of delivery, 8 = miscarriages or induced abortion, 9 = parity, 10 = weight (BMI, gestational weight gain), 11 = psychiatric disorder and/or neurological illness and/or major CNS anomaly and /or intelligence, 12 = use of psychotropic medicine, 13 = pregnancy complications or comorbidity (including gestational diabetes and/or hypertensive diseases and/or systemic inflammatory disease and/or pulmonary disease, heart disease, renal disease, anemia as well as other conditions), 14 = gestational age at delivery, 15 = cohabitation with child’s father at childbirth.

III. Paternal factors: 1 = education, 2 = smoking, 3 = socioeconomic status, 4 = race/ethnicity, 5 = age at time of birth, 6 =BMI, 7 = history of psychiatric disorders, 8 = use of psychotropic medicine.

*ASD* autism spectrum disorder, *ADHD* attention deficit hyperactivity disorder, *BMI* body mass index, *DDs* developmental disorders, *DSM* the diagnostic and statistical manual of mental disorders, *GWG* gestational weight gain, *GDM* gestational diabetes, *ICD* international statistical classification of diseases and related health problems, *IDs* intellectual disabilities, *IQ* intelligence quotient, *PGDM* pre-gestational diabetes, *T1DM* type 1 diabetes, *T2DM* type 2 diabetes.

Studies where the outcomes are compared between siblings discordant for the exposure can enable control of unmeasurable familial confounding as they intrinsically control for maternal genetic factors and environmental exposures that have remained constant or comparable across pregnancies. However, this study design is less applicable where exposure-discordant siblings are scarce, e.g., for exposure being maternal T1DM.

Because of the increasing numbers of pregnant mothers with obesity and/or diabetes, and available preventive and treatments opportunities, it is important to identify the relationship between gestational environment and offspring neuropsychiatric disorders. In this review, we aimed to narratively describe the reported findings about associations of maternal pre-pregnancy obesity and diabetes with offspring neuropsychiatric disorders, and to provide an overview of the plausible underlying mechanisms, as well as challenges for future research.

## Data sources and search strategies

The databases including Web of Science, PubMed and CINAHL were comprehensively searched by using combinations of key words that corresponded to the domains of interest: “offspring OR child*” AND “cognitive development OR cognitive functions OR neurodevelopmental disorders OR neuropsychiatric disorders OR mental health OR psychiatric disorders OR brain development OR ASD OR autism spectrum disorder OR ADHD OR attention-deficit hyperactivity disorder OR schizophrenia OR psychosis OR affective disorder OR behavioral disorders OR intelligence disability OR IQ” AND “mother* OR pregnan* OR women of reproductive age” AND “maternal obesity OR maternal BMI OR Body Mass Index OR adiposity OR overweight OR obesity” AND “type 1 diabetes OR type 2 diabetes OR gestational diabetes OR diabetes insipidus OR diabetes mellitus OR DM OR diabetes”. The publication period interval was from January 1, 2010 to April 1, 2019. This is a narrative review as opposed to a systematic review, which is one of its limitations.

## Autism spectrum disorder (ASD)

### Maternal pre-pregnancy obesity and the risk for offspring ASD

ASD is characterized by developmental delays, communication difficulties, deficits in social functioning, and stereotyped, restricted and repetitive behaviors (American Psychiatric Association, APA) [19]. The association between maternal obesity and offspring ASD has been extensively reviewed confirming an increased risk of ASD in offspring of obese mothers [20–22].

A systematic review and meta-analysis involving 943,293 children and 30,337 cases searched up to March 2018 found that both maternal obesity (OR = 1.41, 95% CI = 1.19–1.67) and maternal overweight (OR = 1.16, 95% CI = 1.05–1.27) were significantly associated with ASD in offspring [23]. Similarly, another meta-analysis including 509,167 participants and 8403 diagnosed cases reported that maternal overweight and obesity was found to have 28 and 36% higher risk for ASD than normal weight during pre-pregnancy or pregnancy [24]. These results are consistent with the other meta-analysis studies [25, 26].

The largest nationwide cohort study including 649,043 births between 2004 and 2014 in Finland showed that obese mothers had 28% increased risk of having a child with ASD compared with mothers with a normal BMI after adjusting for potential confounders (HR = 1.28, 95% CI = 1.06–1.55) [12]. Also, a population-based, case-control study including 1004 children aged 2–5 years in California, USA between 2003 and 2010 showed that mothers with obesity were 60% more likely to have a child with ASD (95% CI = 1.10–2.37) [27]. Moreover, a Swedish population-based cohort study including 333,057 participants born 1984–2007 showed that both maternal and paternal obesity were associated with increased risk of ASD in offspring (OR_maternal_ = 1.94, 95% CI = 1.72–2.17; OR_paternal_ = 1.47, 95% CI = 1.12–1.92) [28].

However, a population-based, prospective Norwegian cohort study including 92,909 children born from 1999 to 2009 did not replicate the maternal obesity-offspring ASD association (OR = 1.09, 95% CI = 0.74–1.59) [29]. Further, the association between maternal obesity and the risk of ASD was not supported by a study using sibling–comparisons [28], which indicated that maternal BMI may be a proxy indicator for familial risk factors such as socio-economic status (SES) and genetic background [28, 30]. However, the aforementioned studies reporting positive association were adjusting for various potential familial factors suggesting that obesity-associated intrauterine environment explains at least some of the maternal obesity-offspring ASD association.

### Maternal diabetes and the risk for offspring ASD

Maternal diabetes was found to be positively associated with ASD risk in offspring, based primarily on studies of GDM. A systematic review and meta-analysis published in 2018 involving 16 studies reported that GDM was associated with ASD in children (RR = 1.48, 95% CI = 1.26–1.75) [16]. Another meta-analysis of mothers with diabetes before or during pregnancy, i.e., not only GDM, involving 12 studies also demonstrated an increased risk of ASD in offspring. The pooled RR was 1.72 (95% CI = 1.24–2.41) among case-control studies, and 1.48 (95% CI = 1.25–1.75) among cohort studies [31].

A large, multiethnic clinical longitudinal cohort study in California including 322,323 singletons born in 1995–2009 showed that the ASD risk in offspring after adjusting for potential confounders was associated with both maternal GDM diagnosed by the 26th gestational week (HR = 1.42, 95% CI = 1.34–2.32) and maternal preexisting T2DM (HR = 1.33, 95% CI = 1.07–1.66) [14]. Similarly, in a prospective national cohort study including 66,445 pregnancies in the USA, GDM was found to be associated with increased risk of ASD (OR = 1.76, 95% CI = 1.34–2.32) [32].

However, the results from other studies investigating the association between diabetes and offspring ASD have been conflicting. For example, a large Finnish nationwide cohort study including 649,043 newborns between 2004 and 2014 did not find any clear effects of neither GDM nor PGDM on ASD risk in normal-weight mothers (OR_GDM _= 1.06, 95% CI = 0.88–1.28; OR_PGDM_ = 0.54, 95% CI = 0.20–1.44) [12]. Another retrospective longitudinal cohort study including all live births (129,733 births) among residents in a Canadian province between 1990 and 2002 found that neither diabetes before the onset of pregnancy nor GDM was associated with ASD (OR_DM_ = 1.98, 95% CI = 0.94–4.16; OR_GDM _= 1.29, 95% CI = 0.90–1.83) [33]. The discrepancy between findings could possibly be influenced by different BMI profiles between different cohorts in mothers with T2DM or GDM and in mothers without diabetes.

### Maternal pre-pregnancy obesity and diabetes in combination and the risk for ASD

The joint effect size between maternal obesity and diabetes on offspring ASD has been reported to be larger than that of obesity or diabetes alone. The Boston birth cohort study between 1998 and 2014 among 2734 children reported three-fold increased risks for ASD of maternal pre-pregnancy obesity and pre-gestational diabetes (PGDM) in combination (HR = 3.91; 95% CI = 1.76–8.68), as well as maternal obesity and GDM in combination (HR = 3.04; 95% CI = 1.21–7.63) [15]. Recently, a Finnish population-based cohort study consisting of 649,043 participants born between 2004 and 2014 showed that ASD risk in offspring was three- to six-fold increased for mothers with insulin-treated PGDM and obesity (HR = 3.64; 95% CI = 1.63–8.16), as well as for mothers with insulin-treated PGDM combined with severe obesity (HR = 6.49; 95% CI = 3.08–13.69) compared to normal-weight nondiabetic mothers [12]. The corresponding joint effects of GDM with obesity and severe obesity were HR = 1.56 (95% CI = 1.26–1.93) and HR = 1.37 (95% CI = 1.04–1.81), respectively [12].

## Attention‐deficit/hyperactivity disorder (ADHD)

### Maternal pre-pregnancy obesity and the risk for offspring ADHD

ADHD is marked by an inattention and/or hyperactivity-impulsivity that interferes with functioning or development. The impact of maternal obesity on offspring ADHD has been reviewed in the past five years. For example, a systematic review and meta-analysis including 41 studies published before April 2017 indicated that maternal pre-pregnancy obesity increased the risk of ADHD in offspring (OR = 1.62, 95% CI = 1.23–2.14) [25].

Recently, a large, population-based nationwide cohort study in Finland consisting of 649,043 births between 2004 and 2014 reported that maternal pre-pregnancy obesity was associated with offspring ADHD after adjusting for potential confounders (OR = 1.44, 95% CI = 1.28–1.63) [12]. Further, a prospective pregnancy cohort from Sweden, Denmark and Finland involving 12,556 school-aged children indicated that obese mothers had an approximately doubled risk of ADHD in offspring (OR = 1.89, 95% CI = 1.13–3.15) [34]. Besides, a longitudinal study reported that maternal pre-pregnancy BMI played a predictive role in the poorer psychosocial development, as evidenced by lower social competence and higher externalizing symptoms in offspring [35].

Additionally, a large population-based cohort study of 673,632 children born in Sweden between 1992 and 2000 showed that maternal obesity was associated with an increased risk of offspring ADHD (HR = 1.64, 95% CI = 1.57–1.73) after adjustment for measured covariates. In full-sibling comparisons of the same cohort, however, the associations no longer remained (HR = 1.15, 95% CI = 0.85–1.56) [36]. Similarly, another population-based sibling-comparison study consisting of 4682 children in the upper Midwest of the USA between 1995 and 2013 found no statistically significant association between maternal obesity and ADHD risk among siblings, although one unit higher BMI increased the risk of offspring ADHD by 4.2% (95% CI = 1.02–1.06) at maternal level [37]. The two sibling-comparison studies suggested that besides the major exposures of interest, other unmeasured familial lifestyle-related characteristics associated with obesity and ADHD may explain part of the maternal obesity–offspring ADHD associations detected [7]. Hence, similar to the case for maternal obesity-offspring ASD association, the sibling analyses do not support an intrauterine environmental causality, however the rigorous adjustments in the other studies for various potential familial factors support some degree of involvement of an obesity-associated intrauterine environment.

### Maternal diabetes and the risk for ADHD

Studies have explored the relationship of both maternal GDM and maternal pre-pregnancy diabetes to the risk of offspring ADHD. A longitudinal cohort study including 212 preschool children with ADHD in New York found that GDM was associated with an over 2-fold increased risk for ADHD (OR = 2.20; 95% CI = 1.00–4.82) in offspring at age 6 years compared with those unexposed [11]. A large population-based cohort study including 649,043 births in Finland between 2004 and 2014 studied normal-weight mothers and found smaller effects of GDM on offspring ADHD risk (HR = 1.15; 95% CI = 1.01–1.30) [12]. Moreover, a retrospective birth cohort study including 333,182 singletons born in 1995–2012 within Kaiser Permanente Southern California hospitals found that the adjusted HRs for offspring ADHD were 1.26 (95% CI = 1.14–1.41) for GDM requiring antidiabetic medication, and 0.93 (95% CI = 0.86–1.01) for GDM not requiring antidiabetic medication, compared with children unexposed in utero to diabetes [13]. Exposure to T1DM had an adjusted HRs for ADHD in children at 1.57 (95% CI = 1.09–2.25), and 1.43 (95% CI = 1.29–1.60) for T2DM [13]. Further, a longitudinal nationwide register-based cohort study involving 2,274,713 in Norway from 1967 to 2012 reported that pre-pregnancy T1DM was associated with an increased risk for ADHD in offspring after adjusting for potential confounders (OR = 1.6; 95% CI = 1.3–2.0), while exposure to maternal T2DM was not associated with offspring ADHD risk (OR = 1.1; 95% CI = 0.7–1.8) [38].

Similarly, maternal pre-pregnancy T1DM was associated with an increased risk of offspring ADHD (RR = 1.31, 95% CI = 1.03–1.63) in a large register-based cohort study including 983,680 individuals born in Denmark from 1990 to 2007 [39],, and in a retrospective cohort study involving 15,615 individuals born in Sweden between 1970 and 2012 (HR 1.35; 95% CI = 1.18–1.55) [40].

Overall, there is support for that exposure to maternal T1DM increases the offspring risk of ADHD modestly. The influence of GDM or T2DM on offspring ADHD risk is less clear. Various maternal and birth factors were adjusted for in aforementioned studies, again proposing some involvement of intrauterine environment, however, sibling pair analyses have not been reported. For T1DM, sibling pair analysis would require very large populations as exposure-discordant siblings are rare.

### Maternal pre-pregnancy obesity and diabetes in combination and the risk for offspring ADHD

Only few studies have considered the combined effects of maternal obesity and diabetes on the risk of ADHD in offspring. Among the two studies on this topic, a prospective birth cohort study including 2,734 mother-child pairs at the Boston Medical Center from 1998 to 2014 reported that there was no association with offspring ADHD, neither for maternal pre-pregnancy obesity–GDM (HR = 1.20, 95% CI = 0.49–2.93), nor for maternal pre-pregnancy obesity–PGDM (HR = 1.06, 95% CI = 0.34–3.36) [15]. Similarly, another population-based nationwide cohort study including 649,043 births in Finland between 2004 and 2014 found no combined association of maternal obesity and insulin-treated PGDM with risk for offspring ADHD compared to normal-weight mothers without PGDM (HR = 1.00, 95% CI = 0.32–3.10) [12]. This study, however, found a markedly higher risk for ADHD for offspring of severely obese mothers with insulin-treated PGDM (HR = 6.03, 95% CI = 3.23–11.24; HR_no diabetes, severe obesity_=1.88, 95% CI = 1.58–2.23). This study also indicated that the joint effect of maternal GDM combined with obesity, or severe obesity, increased the risk for offspring ADHD (HR_GDM, obesity_=1.64, 95% CI = 1.42–1.88) compared with that of mothers with only obesity (HR_no diabetes, obesity_=1.44, 95%CI = 1.28–1.63) [12].

## Cognitive function and intellectual ability

### Maternal pre-pregnancy obesity and the risk for offspring cognitive and intellectual function

The impact of maternal obesity on cognitive function and intelligence development in the offspring has been reviewed [10, 20, 22, 41]. A meta-analysis published in 2018 representing 36 cohorts indicated that maternal pre-pregnancy overweight or obesity was associated with increased risk of offspring developmental delay (OR = 1.58, 95% CI = 1.39–1.79) and emotional/behavioral problems (OR = 1.42; 95% CI = 1.26–1.59) [25]. An increasing number of longitudinal, prospective, and observational studies have explored the association between maternal pre-pregnancy BMI and cognitive performance in the offspring [42–48]. Among these previous studies, most studies suggested that maternal pre-pregnancy obesity was associated with low IQ, including poorer motor, spatial, and verbal skills. [42–45, 48–50] Also a prospective population based cohort of 19,517 children at 5 and 7 years of age in the United Kingdom showed that maternal pre-pregnancy BMI was negatively associated with children’s cognitive performance. The relationship appeared to become stronger as children got older, although the overall effect size was modest [42]. To address potential paternal confounding, a few studies considered not only maternal but also paternal BMI [43, 44, 48]. Wherein, a Danish national birth cohort study including 1783 mothers reported that increased maternal and paternal pre-pregnancy BMI were associated with lower offspring IQ at similar effect sizes after adjusting for potential confounders, suggesting that not only the intrauterine environment was involved [43]. However, findings from two southern European birth cohort studies from Spain and Greece found that maternal BMI effect estimates on infant cognitive development were greater than those of paternal BMI [44], which was consistent with mainly maternal-specific or intrauterine effects. Moreover, one study including two cohorts, one British (*N* = 5000) and the other Dutch (*N* = 2500), showed that neither maternal nor paternal pre-pregnancy overweight were consistently associated with child cognitive abilities [48]. Thus, when interpreting the findings potential unidentified genetic and familial confounding factors and ongoing brain maturation to early adulthood should be considered.

### Maternal diabetes and the risk for offspring cognitive and intellectual function

Maternal PGDM and GDM have been studied as potential factors impairing cognitive function in childhood [51–54]. In particular, a systematic review and meta-analysis including 12 studies and 6140 infants assessed the cognitive abilities in children (up to 14 years) of diabetic and non-diabetic mothers, and found no significant difference [55]. A large family-based prospective cohort study (*N* = 664,871 from 543,203 families) linking nation-wide registers in Sweden reported that maternal GDM was associated with lower educational achievement and IQ scores among men at 16–18 years after adjustment for potential confounders including also maternal pre-pregnancy BMI, while there was no such association within siblings [53]. Thus, this association was likely due to common shared familial characteristics such as familial socioeconomic position, known to influence both educational attainment and GDM, rather than intrauterine mechanism [53].

## Depression, anxiety, psychosis and eating disorders

Depression is characterized by a depressed mood, loss of interest and a feeling of worthlessness, whereas anxiety is characterized by worried thoughts, fear and associated physical symptoms. Only few human studies have investigated if maternal obesity is associated with offspring mood or anxiety disorders, and for maternal diabetes exposure no report on offspring mood and anxiety disorders was found. A population-based cohort study from the Western Australian Pregnancy Cohort including 2,868 live births followed 17 years indicated that maternal pre-pregnancy obesity was associated with childhood affective problems including depression and anxiety (OR = 1.72, 95% CI = 1.11–2.67) [56].

Psychosis is a dissociation from reality based on irregular thoughts and changes of cognitive function. A qualitative review including four studies with 305 cases of schizophrenia and 24,442 controls showed that maternal pre-pregnancy obesity was associated with 2- to 3-fold increased risk of schizophrenia in offspring, although maternal schizophrenia and hence possible genetic confounding was not taken into account [57]. A population-based cohort study of 526,042 individuals born in Sweden between 1982 and 1989 reported a weak U-shaped association between maternal early-pregnancy BMI and the risk for non-affective psychosis in offspring, and the matched-sibling analyses found no association between maternal overweight (HR = 1.11, 95% CI = 0.73–1.68) or obesity (HR = 0.56, 95% CI = 0.23–1.38) and risk for non-affective psychosis in offspring [58]. Eating disorders include anorexia, bulimia and binge-eating disorder. A population-based Australian pregnancy cohort study including 1,383 offspring followed prospectively from 14 to 20 years of age found that maternal early-pregnancy BMI associated positively with the risk of eating disorders in offspring (OR = 1.10, 95% CI = 1.05–1.15) [59].

## Brief overview of potential pathways and mechanisms

**Fig. 1:**
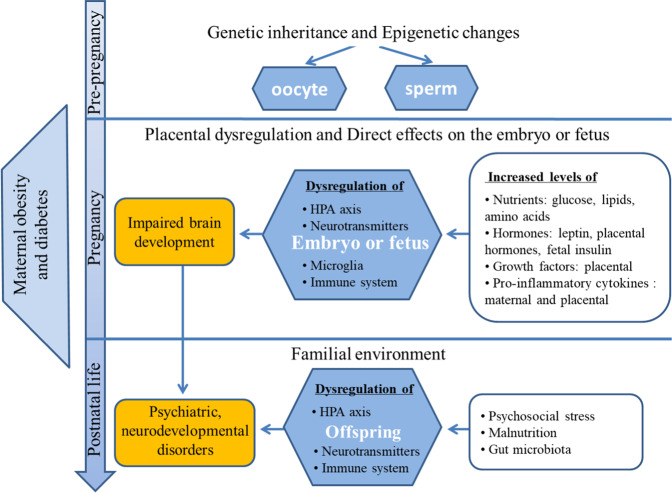
Pathways linking maternal obesity and diabetes with potential neurodevelopmental and psychiatric disorders in offspring. Genetic inheritance, pre-pregnancy and pregnancy effects of a metabolically dysregulated environment, and postnatal effects of psychosocial stress, malnutrition and the microbiota are illustrated.

Confounded by parental genetic and familial postnatal environmental factors, it is challenging to explore underlying mechanisms explaining the putative associations between maternal obesity/diabetes and offspring neurodevelopmental and psychiatric disorders. However, animal models can provide putative causal links between exposure to maternal obesity/diabetes and some behavioral symptoms or functionalities associated with human neurodevelopmental and psychiatric disorders in offspring (Fig. 1). Rodent models on high fat diet (HFD) or with hyperglycemia have been used in this context supporting a role of the intrauterine environment [20, 60–63]. Offspring to pregnant dams of these models display altered behaviors such as hyperactivity, reduced sociability and anxious and depressive-like behaviors [20]. The potential underlying mechanisms between prenatal HFD exposure and offspring neurodevelopment have been reviewed previously [64]. Here, we give a brief overview of the putative key mechanisms of how model and human maternal obesity and diabetes can influence offspring neurodevelopment.

During the human pregnancy, insulin resistance is gradually elevated to ensure adequate nutrition for the fetus. Thus, postprandial glucose levels, basal and stimulated insulin secretion, and hepatic glucose production are elevated. An obese and/or diabetic state can exacerbate the normal pregnancy-related metabolic changes. Insulin resistance, hyperinsulinemia, elevated plasma leptin levels and low-grade inflammation are commonly seen in obese and/or diabetic pregnant mothers [65–69], also in a non-pregnant severely obese women [70]. These metabolic and inflammatory states affect the placenta and thereby influence the exchange of nutrients between the mother and the fetus. Thus, these states leads to increased placental transfer of glucose and fatty acids, placental inflammatory cytokine release and influences the placenta’s production of hormones and growth factors that are crucial for embryonic development [71–74]. The elevated levels of glucose, fatty acids, leptin, inflammatory cytokines and chemokines also directly expose the fetus as these molecules can pass the placenta [75]. Consequently and importantly, fetal secretion of insulin is increased in response to elevated glucose levels passed over from the mother, and so is fetalinsulin resistance [76]. Exposure of the fetus to maternal obesity and diabetes has in rodent models been suggested to have long-lasting effects on organ development and function, including also neuroendocrine regulation and brain development [64], through hyperglycemia [77], oxidative stress [78, 79], lipotoxicity [80, 81], inflammation [82–84] and the associated hormones insulin and leptin [20, 66, 85]. The long-lasting effects can also be mediated by epigenetic modifications.

## Metabolic hormones

As aforementioned, insulin resistance is gradually elevated during pregnancy causing increased plasma insulin levels. An obese and/or diabetic state can exacerbate these pregnancy-induced changes in maternal insulin levels as insulin resistance, hyperinsulinemia and low-grade inflammation are commonly seen in these states [70]. Inflammation, often initiated in white adipose tissue of obese subjects, is known to induce insulin resistance by inhibiting insulin receptor signaling [86]. Insulin resistance in GDM is associated with elevated levels of TNF-α and pro-inflammatory cytokines during pregnancy [87]. Insulin does not pass the placenta, but glucose do, so the fetus becomes exposed to the maternal glycemic state, and in the event of hyperglycemia or fetal insulin resistance it secrets more fetal insulin. Moreover, insulin is an important growth factor in brain development [88]. Increased peripheral insulin levels results in insulin resistance which itself has large effects on the brain and have been associated with depression [89–92].

Leptin is mainly produced by adipose tissue, and leptin receptors are widely distributed in the central nervous system [93]. Leptin resistance is common in an obese state, one potential mechanism being reduced leptin transport across the blood-brain barrier due to elevated levels of triglycerides. Hence, plasma leptin levels are elevated in obesity. Maternal plasma leptin levels are increased during normal pregnancy, likely because of production in the placenta. In obese pregnant women, leptin levels are elevated in plasma, placenta, umbilical cord and fetus compared to non-obese pregnant women. Leptin is produced also by the fetus and is potentially involved in offspring brain development, likely through activation of pro-inflammatory cytokines with downstream effects on neurotransmitters influencing behaviors. Thus, leptin signaling stimulates pro-inflammatory cytokine secretion and influences cortisol release, serotonin and dopaminergic pathways, brain-derived neurotrophic factor (BDNF) signaling and hippocampal synaptic plasticity [93]. Altered leptin levels have been implicated in depression [94]. Elevated early childhood leptin levels in plasma, but not in cord blood, have been associated with an increased risk for autism [95], and associations between leptin levels and other behaviors have been reported from rodent models [93].

Oxytocin is produced primarily by the hypothalamus while its receptors are present both in the nervous system and peripherally, e.g., the adipose tissue. Elevated oxytocin levels influence food intake, adipose tissue metabolism and insulin sensitivity [96, 97]. Oxytocin deficient mice develop late-onset obesity despite normal food intake. Male offspring to pregnant dams on HFD had upregulated oxytocin receptor mRNA through epigenetic changes in the developing hippocampus [98]. Oxytocin modulates a wide range of neurotransmitter and neuromodulator activities and is well known to regulate social behavior, and might be linked to the pathogenesis of neuropsychiatric disorders, such as autism, schizophrenia and eating disorders [99, 100]. Additionally, oxytocin levels were reported to be critical for the developmental GABA switch from being excitatory to being an inhibitory neurotransmitter in rats [101], while lack of this switch induced autism-like behavior.

## Epigenetic effects of maternal obesity and diabetes

The effects of hyperglycemia are in rodent models partly mediated by epigenetic changes, being modifications of histones, DNA and noncoding RNAs ultimately influencing gene expression [102]. Indeed, maternal hyperglycemia is associated with increased DNA methylation in specific genes in offspring, inducing chronic effects for offspring development [103]. Furthermore, methylation-mediated epigenetic mechanisms for intergenerational susceptibility to metabolic disorders were found in a mouse model for GDM [104]. Epigenetic effects of obesity and diabetes on the offspring have in models been shown to occur not only at the fetus level, but also already in the oocyte and sperm [103–106] implying an influence of both maternal and paternal metabolic disorder [103–107]. Reported DNA methylation changes of maternal metabolic state with putative effects in the brain include, but are not limited to, those on offspring leptin signaling [93, 108].

In addition to metabolically induced epigenetic changes in the nuclear genome, the effects of maternal obesity and diabetes on mitochondria and mitochondrial dysfunction [109] might be considered a possible mediator of maternal-specific effects on the offspring, since mitochondrial DNA is inherited from the mother. Mitochondrial dysfunction has been suggested to be a mechanism underlying the pathogenesis of a range of neuropsychiatric disorders such as depression, schizophrenia and bipolar disorder [110].

## Immune activity effects

Low-grade inflammation is commonly seen in obese and/or diabetic pregnant mothers. Pro-inflammatory cytokines passing the placenta to the fetus exposing the offspring brain during gestation are known to influence the development of neural pathways regulating behavior, such as the hypothalamic-pituitary-adrenal (HPA) axis, and serotonergic and dopaminergic systems and BDNF levels [60]. IL-6 is an early key pro-inflammatory cytokine. A recent longitudinal study found that maternal IL-6 concentrations during pregnancy were associated with neonate differences in functional brain networks for social, emotional and cognitive development, and subsequently in concert with working memory performance in 2-year-old offspring [111]. Higher levels of IL-6 may lead to offspring cognitive and behavioral deficits by altering the formation of synapses [112]. Pro-inflammatory cytokines, as well as BDNF, influence the differentiation and survival of neurons, synaptic plasticity and hence functional connectivity, essential for neurodevelopment and behavior, where also microglia play a key role [113, 114].

Microglia are motile myeloid cells in the brain, which play a critical role in neurodevelopment through regulating both neurogenesis and pruning, besides having traditional macrophage-type roles. Excess exposure to pro-inflammatory cytokines in the developing brain induces improper activation of microglia, resulting in improper support and regulation of neuronal activity, impeding neuron differentiation and survival [113, 115, 116]. Rodent studies showed that dysregulation of microglia is associated with autism and schizophrenia [117], and microglia play an active part in obesity-related cognitive decline by phagocytosis of synapses [118].

## Glucocorticoids and the hypothalamic-pituitary-adrenal (HPA) axis

Inflammation is known to upregulate the HPA axis [119]. The HPA axis regulates the neuroendocrine stress response through circulating cortisol, or in rodent corticosterone. A long-term overactive HPA axis increases the risk for impaired hippocampal glucocorticoid receptor (GR) feedback, elevated stress susceptibility and depressive and anxiety phenotypes. In mice and rats, maternal HFD has been reported to lead to increased maternal levels of corticosterone, which under this diet more readily passed through the placenta to the fetus [120]. The offspring had long-term elevated corticosterone levels, a dysregulated HPA-axis and were more sensitive to stressors with amygdala playing a role [121]. In humans, higher maternal early pregnancy BMI was reported to be associated with lower morning salivary cortisol in the offspring at adult age [122]. The placenta is a main source of corticotrophin releasing factor (CRF) [123], which feeds back to both the fetal and maternal pituitary to secrete ACTH stimulating cortisol/corticosterone production [124, 125]. Placental CRF plays a crucial role in regulating the fetal HPA axis [124, 126]. In rodent studies, the reduced HPA axis sensitivity and anxiety-like behaviors in offspring of dams with overactive HPA axis during pregnancy was in part explained by an attenuated hippocampal GR expression [127, 128]. Also immune activity and psychosocial stress causes elevated glucocorticoid levels [129]. Maternal psychosocial stress is known to long-term modify the offspring HPA axis activity epigenetically towards increased stress sensitivity [130] and suicide risk [131, 132].

## Serotonergic and dopaminergic system effects

Changes in development of the serotonergic system and in serotonin signaling have been observed in rodent and non-human primate models owing to maternal inflammation or a HFD, leading to increased risk for behavioral abnormalities in offspring [133–135]. Altered dopamine sensitivity, as well as abnormal dopamine and *BDNF* expression are also documented in rats of mothers fed with HFD [60, 136, 137]. Moreover, maternal inflammation, modeled with IL-6 and leptin, has been associated with dopamine signaling in offspring [138]. Serotonin and dopamine are known key neurotransmitters in psychopathology, and have together with BDNF been suggested by a growing body of evidence to also be linked to the pathogenesis of several neuropsychiatric disorders [99, 139].

## The gut-brain axis

Another putative link from maternal obesity and diabetes to offspring neuropsychiatric disorders is a putative maternal gut-fetal brain axis. HFD dramatically alters the intestinal microbiota, and the gut bacterial microbiota is less diverse and more inflammatory in persons with obesity or diabetes [140]. A less diverse gut microbiota has been reported also in psychiatric patients, and feces from patients with ASD, depression or schizophrenia transferred to the rodent intestine produced disorder-related behaviors and biochemical modulations [141–143]. The gut microbiota communicates with the enteric nervous system in the gastrointestinal organs, which signals via vagus and ganglia to the central nervous system. In fact, gut bacteria show species-specific production of neuroactive substances, e.g., dopamine, serotonin and GABA neurotransmitters. Other key routes of the gut-brain axis include the bacterial fermentation metabolites short chain fatty acids (SCFAs), and immune activation, both regulating the function of the intestinal and blood-brain barriers [144]. The SCFAs pass the blood-brain barrier and have broad effects on nervous system physiology such as mitochondrial function and microglial maturation and activation [145, 146]. SCFAs at physiological levels regulate early growth and proliferation of human neural proliferating cells [147]. Thus, gut microbiota metabolites, such as SCFAs, might have an effect on the offspring neurodevelopment and hence mediate a putative maternal-gut fetal-brain axis. Also, a maternal gut bacterial flora activating Th17-cell has been reported to increase the risk of rodent offspring behavior and brain structure abnormalities resembling ASD after exposure to maternal immune activation, such as infection [148]. Both disruptions in neuronal architecture by direct intracerebral action through interleukin-17a pathway, and altered signatures of gut microbiota have been documented in offspring with maternal immune activation [72].

## Genetic factors

As a result of the altered intrauterine environment, as aforementioned, neurogenesis/apoptosis, synaptic pruning, neuronal migration, and neuronal connection could be affected [149, 150]. The symptom and functionality outcomes might also in part depend on factors such as genetic and postnatal factors. Obesity and type 2 diabetes are known to be heritable, the inherited susceptibility of which relate to accumulation of many common DNA variants [151]. Recent genome-wide-association-studies (GWAS) revealed that more than 200 loci associated with type 2 diabetes and obesity traits [152–154]. Neurodevelopmental and psychiatric disorders are also moderately (depressive, anxiety, eating and sleeping disorders) to highly (ASD, AHDH, bipolar and psychotic disorders) heritable [155]. Notably, children with ADHD or ASD have been reported to have an enhanced risk for developing obesity [156, 157]. One possibility is that obesity and neurodevelopmental disorders share some common genetic or metabolic pathways. GWAS loci associated with obesity have been shown to be nearby genes involved in appetite, energy homeostasis and mood regulation [158–160]. Additionally, the gene for β2-Adrenoceptor (ADRB2, a G protein-coupled receptor) was involved in the circulatory, muscle, and digestive system. This gene was also linked to insulin resistance [161], obesity/diabetes [162, 163], and psychiatric disorders such as autism [164].

Finally, maternal pre-pregnancy obesity and diabetes seem to jointly potentiate their offspring to adverse neurodevelopment [12, 165, 166]. The timing of obesity and/or diabetes onset might be important in determining the offspring neurodevelopmental outcomes. The most vulnerable period would be peri-conceptional and early pregnancy, during which period the epigenetic programming, placentation and fetal brain organogenesis happens [167]. Environmental exposures during postnatal life such as familial parenting style may also influence the offspring neurodevelopment [168].

## Challenges for future research

The extensive adjustment for potential confounders in previous studies might induce underestimation of effect sizes. On the other hand, sibling pair analyses suggest the influence of elusive familial factors on the association between maternal obesity and offspring ASD and ADHD. Thus, the causality of the observed associations remains unclear. Further, ASD and ADHD often co-occur. Future studies should tease out common and unique factors contributing to these disorders. Still, experimental research in rodents clearly supports a causal effect of a dysmetabolic milieu, for germ cells and fetus, resulting in phenotypic effects resembling some characteristics of pediatric neurodevelopmental and psychiatric disorders. Hence, alongside further experimental mechanistic research, large sophisticated prospective studies controlling for familial confounding are needed to obtain further insights into the multiple effectors and underlying mechanisms of the observed associations, such as sibling-comparison and maternal-paternal comparison studies and Mendelian randomization studies. Future studies should carefully consider gene × environmental interactions, which are likely underlying most of the neurodevelopmental disorders, hence exposure and diagnoses outcome data should be combined with multi-omics profiling (genome, epigenome, metabolome). Especially, sibling pair analyses (including twin pairs) combined with multi-omics profiling (genome, epigenome, metabolome) would be powerful in delineating specific familial factors. Here, detailed information and assessments are important, such as repeated measurements of maternal BMI, body composition and metabolic status from first trimester and onwards, as well as specific maternal lifestyle and nutritional factors that might strengthen effects of maternal obesity and diabetes on offspring neuropsychiatric disorders. Finally, although specific dietary interventions exist, such as omega-3 supplementation, there is a lack of innovative and effective intervention research starting in the first trimester with long-term follow-up of offspring regarding the moderation of maternal metabolic exposures, before or during pregnancy, on the risk of offspring neurodevelopmental or psychiatric disorders [169].

## Concluding remarks

This narrative review highlights previous research regarding associations of maternal obesity and diabetes with neurodevelopmental and psychiatric disorders in offspring. The associations between maternal obesity on the one hand, and offspring ASD, ADHD and cognitive function on the other hand, have been extensively studied in large cohorts with results displaying modest effect sizes after adjustment for confounding maternal and birth factors. Those effects are reported to likely be explained in part by an obesity-associated intrauterine milieu, and in part by maternal genetic background and other familial factors. The importance of the latter factors was concluded through analyses applying a sibling analysis design or a paternal-maternal-comparison. The influence of maternal obesity on other psychiatric disorders is less well studied, but there are reports of associations with increased risk for offspring depression, anxiety, schizophrenia and eating disorders, at modest effect sizes. The effect of maternal T2DM and GDM on offspring ASD, and that of maternal T1DM and GDM on offspring ADHD have been explored, again revealing modest positive effect sizes. Here, there are no reports of sibling analyses or maternal-paternal comparisons. Notably, a few studies have reported larger effect sizes on risks for ASD and ADHD for mothers with both obesity and diabetes, which might reflect a more metabolically impaired intrauterine milieu. This review also provides an overview of plausible underlying mechanisms, which support both an actual involvement of a metabolically dysregulated intrauterine milieu on neurodevelopment, and familial factors, although interpretation of rodent model findings into the human case must be done with caution. The current knowledge on effectors in the intrauterine milieu, which is based primarily on rodent models, proposes a role of particularly hyperglycemia-induced epigenetic effects and pro-inflammatory cytokines. Prospective maternal-paternal comparison and sibling-comparison studies, experimental animal model studies and randomized controlled trials are required to examine the causality, underlying mechanisms and the potential for prevention of maternal metabolic exposures.
